# Dehydration in cerebral venous sinus thrombosis

**DOI:** 10.1111/cns.14760

**Published:** 2024-05-22

**Authors:** Jiayue Ding, Duo Lan, Xiaoming Zhang, Xiangyu Li, Yanli Duan, Xiaobing Tian, Zhangyuan Liao, Xuanye Yue, Ming Zou, Ran Meng

**Affiliations:** ^1^ Department of Neurology Tianjin Medical University General Hospital Tianjin China; ^2^ Department of Neurology, Xuanwu Hospital Capital Medical University Beijing China; ^3^ Department of Neurology Tianjin Huanhu Hospital Tianjin China

**Keywords:** cerebral venous sinus thrombosis, dehydration, prognosis, urine specific gravity

## Abstract

**Aims:**

This study aimed to unravel the dehydration status of patients with cerebral venous sinus thrombosis (CVST) to facilitate the understanding of dehydration in CVST.

**Methods:**

This was a multicenter retrospective study and three populations were recruited, namely, patients with CVST, CVST mimics, and healthy subjects. Blood samples were obtained 1–2 days after admission to assess dehydration status. Stata 15.1 was performed for statistical analysis.

**Results:**

A total of 208 patients were diagnosed with CVST, 237 with CVST mimics, and 200 healthy individuals were enrolled. The urine specific gravity (USG, 1.020 [1.014, 1.029] vs. 1.017 [1.011, 1.021]) was higher in patients with CVST than in those with mimics (all *p* < 0.001). The percentage of USG >1.03 was also higher in CVST (22.6%) than in its mimics (6.3%, *p* < 0.001). With the development of CVST, USG (acute vs. sub‐acute vs. chronic, 1.022 [1.015, 1.033] vs. 1.021 [1.015, 1.031] vs. 1.019 [1.014, 1.025]) decreased. All dehydration‐related markers could not differentiate CVST from its mimics and healthy populations, and they were not associated with CVST severity and prognosis (*p* > 0.05).

**Conclusion:**

High levels of USG, especially USG >1.013, were more common in patients with CVST. Dehydration‐related indices could not characterize CVST and were not associated with CVST severity and prognosis.

## Graphical abstract

This study found that high levels of urine specific gravity (USG), especially USG >1.013, were more common in patients with cerebral venous sinus thrombosis (CVST). With the development of CVST, USG decreased. Dehydration‐related indices could not characterize CVST and were not associated with CVST severity and prognosis.

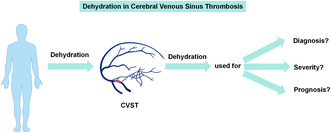

## 1 INTRODUCTION

Cerebral venous sinus thrombosis (CVST) is an uncommon but life‐threatening stroke subtype occurring primarily in young and middle‐aged individuals.^1^, ^2^ Many risk factors are associated with CVST, including obstetric causes, infection, anemia, oral contraceptive use, and autoimmunity diseases.^3^ The main pathogenesis of these risk factors is a hypercoagulable state, which induces thrombus formation and is reflected by a high D‐dimer level.^4^ Theoretically, dehydration is related to a hypercoagulable state because it can reduce regional blood flow and increase blood viscosity.^5^, ^6^, ^7^ The relationship between dehydration and acute ischemic stroke (AIS) has been investigated extensively.^7^ Dehydration‐related biomarkers, such as the urea (UA)/creatinine (Cr) ratio, blood urea nitrogen/Cr ratio, urine specific gravity (USG), hematocrit, and plasma osmolality, are always used to reflect the dehydration status, and all of them are linked to the severity and prognosis of patients with AIS.^7^, ^8^, ^9^ Therefore, we hypothesized that these markers would be abnormal in patients with CVST. However, investigations on the relationship between dehydration and CVST are still lacking. Accordingly, this study aimed to unravel the dehydration status of patients with CVST to facilitate the understanding of dehydration in CVST and provide novel insights into the risk factors, diagnostic methods, and therapeutic paradigms for CVST in the future.

## 2 METHODS

### 2.1 Study population

This was a multicenter retrospective observational study conducted in the neurology department of Tianjin Medical University General Hospital, Xuanwu Hospital Capital Medical University, and Tianjin Huanhu Hospital. This study was conducted in accordance with the guidelines of the 1964 Declaration of Helsinki, and all procedures performed in this study involving human participants were approved by the Institutional Ethics Committee. A total of three types of populations were recruited between April 2018 to September 2023, namely, patients with CVST, CVST mimics, and healthy subjects. All the enrolled patients were assessed with magnetic resonance venography (MRV) and/or computed tomography venography (CTV) and confirmed CVST or CVST mimics using the magnetic resonance black‐blood thrombus imaging (MRBTI) technique or digital subtraction angiography (DSA), which can visualize the thrombus directly.^10^ According to the time of symptom onset, CVST was divided into acute (≤15 days), sub‐acute (15–30 days), and chronic (>30 days) stages. Patients with CVST mimics had symptoms similar to definite CVST (such as headache, vomiting, and intracranial hypertension), and these symptoms might be attributed to the anatomic variants of the sinus (such as unexplained stenosis, giant arachnoid granules, idiopathic intracranial hypertension, and nondominant transverse or sigmoid sinus).^10^, ^11^, ^12^ In other words, CVST mimics could be also termed as nonthrombotic cerebral venous sinus stenosis. Patients were excluded if they had (1) other austere diseases; (2) a history of stroke except CVST; and (3) renal dysfunction. People who received physical examination and were without a history of renal dysfunction and severe diseases (such as stroke, cancer, and trauma) were enrolled as healthy subjects. Some chronic disorders, such as hypertension, diabetes, and hyperuricemia, were present in healthy subjects.

### 2.2 Data collection

Clinical data, including patient demographics, symptoms, imaging presentations, risk factors for CVST, and causes of CVST mimics, were comprehensively recorded. The severity and prognosis of CVST were evaluated using the National Institutes of Health Stroke Scale (NIHSS) and the Modified Rankin Score (mRS) at admission and follow‐up. Blood samples were obtained 1–2 days after admission to assess dehydration status, including Cr, urea (UA), USG, hematocrit, blood sodium (Na^+^), blood potassium (K^+^), glucose, uric acid, D‐dimer, and fibrinogen. UA/Cr and plasma osmolality were also calculated to evaluate dehydration. Plasma osmolality was estimated using the following equation: [2 × (Na^+^ + K^+^) + glucose + UA]. Because the enrolled patients did not have renal diseases or renal dysfunction, the dehydration‐related indices assessed in this study mainly reflected blood volume and nutritional status. Peripheral blood collected from healthy subjects only included Cr, UA, hematocrit, glucose, uric acid, and fibrinogen because of incomplete examination.

### 2.3 Statistical analysis

Stata 15.1 was performed for statistical analysis in this study. The Kruskal–Wallis test was used to assess the data distribution. Continuous variables following the Gaussian distribution were displayed as mean ± standard deviation (SD) and analyzed using student's *t*‐test; otherwise, they were presented as median [interquartile range, IQR] and analyzed using the Mann–Whitney U test. Categorical variables were depicted as numbers (percentages) and analyzed using a chi‐square test. Receiver operating characteristic (ROC) analysis was used to demonstrate the sensitivity and specificity of the variables for differentiating patients with CVST from its mimic and healthy subjects. An area under the curve (AUC) >0.6 with a *p*‐value <0.05, was considered significant. The diagnostic power was also assessed using the positive predictive value (+PV), negative predictive value (−PV), positive likelihood ratio (+LR), and negative likelihood ratio (−LR). The optimal cutoff values were confirmed with maximal sensitivity and specificity. Propensity score matching was performed to eliminate confounding factors. Spearman's rank correlation analysis was performed to identify the association between the biomarkers and CVST severity and prognosis. Missing variables were replaced with mean values (continuous variables) or modes (dichotomous variables) only if they accounted for less than 10% of the total. Statistical significance was set at *p* < 0.05. However, the significant level α was adjusted using the Bonferroni method to avoid type I errors when analyzing repeated measurement data.

## 3 RESULTS

### 3.1 Patients

A total of 208 patients were diagnosed with CVST (mean age, 40.65 ± 13.74 years; female, 45.2%), 237 with CVST mimics (mean age, 50.06 ± 16.00 years; female, 77.2%), and 200 healthy individuals (mean age, 42.33 ± 7.92; female, 56%) were enrolled in this retrospective study. The prevalence of symptoms including headache (CVST vs. CVST mimics, 88.9% vs. 44.3%, *p* < 0.001), nausea or vomiting (47.1% vs. 16.5%, *p* < 0.001), neurological deficits (27.4% vs. 7.2%, *p* < 0.001), diplopia (10.6% vs. 4.2%, *p* = 0.010), seizures (20.2% vs. 0.4%, *p* < 0.001) and unconsciousness (9.6% vs. 0.0%, *p* < 0.001) in patients with CVST were significantly higher than that in patients with CVST mimics; however, the symptoms of dizziness (20.7% vs. 39.2%, *p* < 0.001) and tinnitus (15.9% vs. 40.9%, *p* < 0.001) were more commonly seen in patients with CVST mimics. Furthermore, imaging showed that the right transverse sinus (50.0% vs. 17.3%, *p* < 0.001), superior sagittal sinus (58.2% vs. 2.5%, *p* < 0.001) and straight sinus (18.3% vs. 1.3%, *p* < 0.001) were more commonly seen in CVST than in its mimics, and the left transverse sinus (50.0% vs. 73.8%, *p* < 0.001) was more commonly seen in mimics than in CVST. The most common risk factor for CVST was obstetric causes, followed by anemia and oral contraceptive use. The causes of CVST mimics included sinus‐filling defects, sinus atresia/hypoplasia, and giant arachnoid granules. All aforementioned are shown in Table 1.

**TABLE 1:** Characteristics of the enrolled cases.

Characteristics	CVST	CVST mimics	*p*‐value
Demographics			
No. of subjects	208	237	‐
Female, *n*(%)	94 (45.2)	183 (77.2)	<0.001
Age, yr	40.65 ± 13.74	50.06 ± 16.00	<0.001
Clinical manifestation, *n* (%)			
Headache	185 (88.9)	105 (44.3)	<0.001
Nausea or vomiting	98 (47.1)	39 (16.5)	<0.001
Dizziness	43 (20.7)	93 (39.2)	<0.001
Tinnitus	33 (15.9)	97 (40.9)	<0.001
Neurological deficits	57 (27.4)	17 (7.2)	<0.001
Visual disorder	71 (34.1)	81 (34.2)	0.993
Diplopia	22 (10.6)	10 (4.2)	0.010
Seizures	42 (20.2)	1 (0.4)	<0.001
Unconsciousness	20 (9.6)	0 (0.0)	‐
Cerebral hernia	1 (0.5)	0 (0.0)	‐
Involved sinus, *n* (%)			
Right transverse sinus	114 (54.8)	125 (52.7)	0.663
Left transverse sinus	104 (50.0)	175 (73.8)	<0.001
Right sigmoid sinus	104 (50.0)	41 (17.3)	<0.001
Left sigmoid sinus	92 (44.2)	89 (37.6)	0.153
Superior sagittal sinus	121 (58.2)	6 (2.5)	<0.001
Straight sinus	38 (18.3)	3 (1.3)	<0.001
Cortical vein	45 (21.6)	0 (0.0)	‐
Risk factors, *n* (%)			
Infection	54 (26.0)	‐	‐
Obstetric cause	20 (9.6)	‐	‐
Anemia	32 (15.4)	‐	‐
Oral contraceptive uses	11 (5.3)	‐	‐
Causes of mimics, *n* (%)			
Giant arachnoid granules	‐	91 (38.4)	‐
Sinus atresia/hypoplasia	‐	97 (40.9)	‐
Sinus filling defects	‐	73 (30.8)	‐

*Note*: Causes of mimics were verified by MRBTI.

### 3.2 Differences in dehydration‐related indices among CVST, CVST mimics, and healthy subjects

Biomarkers reflecting dehydration status were recorded in this study. According to the Bonferroni method, the significance level α (α = 0.05) was adjusted to 0.003 (0.05/15, for a total of 15 comparisons) to avoid type I errors. We found that the USG (1.020 [1.014, 1.029] vs. 1.017 [1.011, 1.021]) and D‐dimer concentrations (0.360 [0.190, 1.150] vs. 0.240 [0.160, 0.370]) were significantly higher in patients with CVST than in those with mimics (all *p* < 0.001). Moreover, the percentage of USG >1.03 was also higher in CVST (22.6%) than in its mimics (6.3%, *p* < 0.001). Patients with CVST had the lowest UA concentrations among the three cohorts(CVST vs. mimics vs. health subjects, 3.72 [2.93, 4.49] vs. 4.22 [3.38, 5.09] vs. 4.20 [3.60, 5.00], *p* < 0.001). The UA/Cr ratio in patients with CVST mimics (0.073 [0.061, 0.090]) was higher than that in patients with CVST (0.063 [0.050, 0.079], *p* < 0.001) and healthy subjects (0.066 [0.056, 0.081], *p* < 0.001). Despite the small differences, the plasma osmolality and the concentration of Na^+^ were also higher in patients with CVST mimics than in those with CVST (299.48 [296.36, 302.24] vs. 297.15 [293.56, 299.70], *p* < 0.001; 141.00 [140.00, 143.00] vs. 140.00 [138.00, 142.00], *p* < 0.001). The Cr concentrations and hematocrit levels and the number of patients with hematocrit levels >45% were substantially higher in the healthy group (63.00 [54.00, 76.00]; 43.85 [39.73, 47.08]; 41.5%) than in the CVST (57.00 [47.00, 68.75]; 40.25 [36.70, 43.98]; 16.8%, all *p* < 0.001) and its mimics groups (55.00 [48.00, 66.00]; 39.00 [37.20, 41.80]; 11.0%, all *p* < 0.001). In contrast, the fibrinogen concentration was the lowest in healthy subjects (CVST vs. mimics vs. health subjects, 3.03 [2.41, 3.82] vs. 2.97 [2.58, 3.28] vs. 2.75 [2.49, 3.05], *p* < 0.001). Other markers such as K^+^, glucose, and uric acid did not differ among the three groups. All aforementioned are shown in Table 2.

**TABLE 2:** Differences of dehydration‐related indices among patients with CVST, patients with CVST mimics, and health subjects.

Indices	CVST (208)	CVST mimics (237)	Healthy people (200)	*p*‐value^1^	*p*‐value^2^	*p*‐value^3^
Cr	57.00 [47.00, 68.75]	55.00 [48.00, 66.00]	63.00 [54.00, 76.00]	0.725	<0.001	<0.001
Urea	3.72 [2.93, 4.49]	4.22 [3.38, 5.09]	4.20 [3.60, 5.00]	<0.001	<0.001	0.638
USG	1.020 [1.014, 1.029]	1.017 [1.011, 1.021]	‐	<0.001	‐	‐
Hematocrit, %	40.25 [36.70, 43.98]	39.00 [37.20, 41.80]	43.85 [39.73, 47.08]	0.126	<0.001	<0.001
Na^+^	140.00 [138.00, 142.00]	141.00 [140.00, 143.00]	‐	<0.001	‐	‐
K^+^	3.84 ± 0.33	3.89 ± 0.28	‐	0.094	‐	‐
Glucose	4.65 [4.26, 5.17]	4.66 [4.34, 5.04]	4.80 [4.60, 5.10]	0.874	0.027	0.004
Uric acid	300.50 [239.25, 380.00]	304.00 [260.50, 368.50]	326.50 [269.00, 401.50]	0.392	0.016	0.066
UA/Cr	0.063 [0.050, 0.079]	0.073 [0.061, 0.090]	0.066 [0.056, 0.081]	<0.001	0.113	<0.001
D‐dimer	0.360 [0.190, 1.150]	0.240 [0.160, 0.370]	‐	<0.001	‐	‐
Fibrinogen	3.03 [2.41, 3.82]	2.97 [2.58, 3.28]	2.75 [2.49, 3.05]	0.233	<0.001	<0.001
Plasma osmolality, mOsm/(kg·H_2_O)	297.15 [293.56, 299.70]	299.48 [296.36, 302.24]	‐	<0.001	‐	‐
USG >1.03, *n* (%)	47 (22.6)	15 (6.3)	‐	<0.001	‐	‐
Hematocrit >45%, *n* (%)	35 (16.8)	26 (11.0)	83 (41.5)	0.073	<0.001	<0.001
Plasma osmolality >310 mOsm/(kg·H_2_O)	1 (0.5)	2 (0.8)	‐	0.910	‐	‐

*Note*: *p* < 0.002 indicates significance. ^1^CVST vs CVSS; ^2^CVST vs Health people; ^3^CVSS vs Healthy people. *p*‐value <0.003 indicates statistical significance.

Abbreviations: Cr, creatinine; UA, uric acid; USG, Urine specific gravity.

Propensity score matching was performed in this study in order to rule out confounding factors (sex and age). A *p*‐value <0.003 indicated statistical significance. A total of 140 patients with CVST and 140 with CVST mimics were enrolled in this study. The two populations had the same proportions of patients in terms of age and sex. We found that patients with CVST had lower UA (3.60 [2.72, 4.37] vs. 4.00 [3.27, 4.97], *p* = 0.001) and Na^+^ concentrations (140.00 [138.00, 142.00] vs. 141.00 [140.00, 142.00], *p* < 0.001), lower plasma osmolality (297.11 [292.57, 299.68] vs. 299.04 [295.45, 301.54], *p* < 0.001), and higher D‐dimer levels (0.380 [0.210, 1.145] vs. 0.210 [0.140, 0.350], *p* < 0.001) than those with CVST mimics. The percentage of patients with USG >1.03 was significantly higher in the CVST group (25.0%) than in the CVST mimic group (10%). The healthy subject group had higher concentrations of Cr, hematocrit levels, and number of patients with hematocrit levels >45% than the CVST (53.00 [44.00, 65.00]; 38.60 [35.53, 42.18]; 12.1%) and mimic group (57.00 [49.00, 69.00]; 39.25 [37.23, 42.85]; 10.0%). These results are shown in Table 3.

**TABLE 3:** Differences of dehydration‐related indices among patients with CVST, patients with CVST mimics, and health subjects after propensity score matching.

Indices	CVST (140)	CVST mimics (140)	*p*‐value^1^	*p*‐value^2^	*p*‐value^3^
Cr	53.00 [44.00, 65.00]	57.00 [49.00, 69.00]	0.012	<0.001	0.001
Urea	3.60 [2.72, 4.37]	4.00 [3.27, 4.97]	0.001	<0.001	0.185
USG	1.021 [1.014, 1.031]	1.019 [1.014, 1.024]	0.050	‐	‐
Hematocrit, %	38.60 [35.53, 42.18]	39.25 [37.23, 42.85]	0.067	<0.001	<0.001
Na^+^	140.00 [138.00, 142.00]	141.00 [140.00, 142.00]	<0.001	‐	‐
K^+^	3.83 ± 0.33	3.88 ± 0.25	0.103	‐	‐
Glucose	4.68 [4.28, 5.25]	4.53 [4.30, 4.90]	0.069	0.081	<0.001
Uric acid	288.50 [224.25, 370.00]	308.50 [263.00, 382.00]	0.003	<0.001	0.522
UA/Cr	0.064 [0.051, 0.081]	0.070 [0.058, 0.082]	0.093	0.428	0.185
D‐dimer	0.380 [0.210, 1.145]	0.210 [0.140, 0.350]	<0.001	‐	‐
Fibrinogen	3.03 [2.40, 3.71]	2.87 [2.48, 3.28]	0.121	0.001	0.033
Plasma osmolality, mOsm/(kg·H_2_O)	297.11 [292.57, 299.68]	299.04 [295.45, 301.54]	<0.001	‐	‐
USG >1.03, *n* (%)	35 (25.0)	14 (10.0)	0.001	‐	‐
Hematocrit >45%, *n* (%)	17 (12.1)	22 (15.7)	0.388	<0.001	<0.001
Plasma osmolality >310 mOsm/(kg·H_2_O)	1 (0.7)	0 (0.0)	1.000	‐	‐

*Note*: *p* < 0.002 indicates significance. ^1^CVST vs CVSS; ^2^CVST vs Health people; ^3^CVSS vs Health people. *p*‐value <0.003 indicates statistical significance.

Abbreviations: Cr, creatinine; UA, uric acid; USG, Urine specific gravity.

### 3.3 Dehydration‐related indices in patients with CVST at different stages

We set onset‐to‐door time ≤15 days as an acute stage, 16–30 days as the sub‐acute stage, and > 30 days as a chronic stage. There were 62 patients with CVST in the acute stage (7^4^, ^5^, ^6^, ^7^, ^8^, ^9^, ^10^, ^11^, ^12^ days), 43 in the subacute stage (26 [20–30] days), and 103 in the chronic stage (22 [61–365] days). With the development of CVST, some indices, including USG (acute vs. sub‐acute vs. chronic, 1.022 [1.015, 1.033] vs. 1.021 [1.015, 1.031] vs. 1.019 [1.014, 1.025]), D‐dimer (0.970 [0.450, 2.310] vs. 0.380 [0.200, 1.250] vs. 0.220 [0.160, 0.410]), and fibrinogen (3.56 [2.88, 4.27] vs. 3.26 [2.46, 3.80] vs. 2.77 [2.29, 3.43]), decreased, and plasma osmolality (295.84 ± 6.08 vs. 296.68 ± 5.57 vs. 297.37 ± 4.83) increased gradually, all of which gradually reached levels close to those in patients with its mimics. The percentage of patients with CVST with USG >1.03 also decreased as the onset‐to‐door time increased. A comparison of the dehydration‐related indices at different stages of CVST with CVST mimics is presented in Table 4.

**TABLE 4:** Comparison of dehydration‐related indices in acute, sub‐acute, and chronic stage of CVST with CVST mimics.

Indices^#^	Acute (62)	Sub‐acute (43)	Chronic (103)
	7 [4–12] days	26 [20–30] days	122 [61–365] days
Cr	60.25 [50.63, 71.50]	56.00 [44.00, 72.00]^#^	54.00 [46.00, 65.00]^###^
Urea	3.74 [2.72, 4.32]**^/##^	3.54 [2.51, 4.49]**^/###^	3.86 [3.13, 4.50]**^/##^
USG	1.022 [1.015, 1.033]***	1.021 [1.015, 1.031]***	1.019 [1.014, 1.025]**
Hematocrit, %	40.85 [34.18, 44.13]^###^	39.50 [37.80, 44.10]^###^	40.30 [37.40, 43.80]^###^
Na^+^	140.00 [138.00, 142.00]***	140.00 [139.00, 142.00]*	140.00 [139.00, 142.00]**
K^+^	3.82 ± 0.37	3.86 ± 0.33	3.84 ± 0.31
Glucose	4.99 [4.59, 5.52]**^/#^	4.61 [4.20, 4.99]	4.54 [4.17, 4.91]*^/###^
Uric acid	312.22 ± 120.26	288.21 ± 103.53*^/##^	315.73 ± 92.90
UA/Cr	0.060 [0.044, 0.078]***^/#^	0.057 [0.048, 0.073]***^/#^	0.067 [0.055, 0.084]*
D‐dimer	0.970 [0.450, 2.310]***	0.380 [0.200, 1.250]***	0.220 [0.160, 0.410]
Fibrinogen	3.56 [2.88, 4.27]***^/###^	3.26 [2.46, 3.80]^#^	2.77 [2.29, 3.43]
Plasma osmolality, mOsm/(kg·H_2_O)	295.84 ± 6.08***	296.68 ± 5.57**	297.37 ± 4.83**
USG >1.03, *n* (%)	19 (30.6)***	11 (25.6)***	17 (16.5)**
Hematocrit >45%, *n* (%)	11 (17.7)^###^	8 (18.6)^##^	16 (15.5)^###^
Plasma osmolality >310 mOsm/(kg·H_2_O)	1 (1.6)	0 (0.0)	0 (0.0)

*Note*: Compare acute, sub‐acute and chronic stage of CVST with mimics respectively, * < 0.05, ** < 0.01, *** < 0.001; compare acute, sub‐acute and chronic stage of CVST with health subjects respectively, ^#^ < 0.05, ^##^ < 0.01, ^###^ < 0.001.

Abbreviations: Cr, creatinine; UA, uric acid; USG, Urine specific gravity.

### 3.4 ROC analysis for differentiating CVST from CVST mimics and health subjects

Based on comparisons among patients with CVST, CVST mimics, and healthy subjects, parameters that had between‐group differences with a *p*‐value of <0.003 were included in the ROC analysis. As for differentiating CVST from mimics (Table S1), the parameters with AUC >0.6 included USG (AUC, 95%CI: 0.637, 0.585–0.688, *p* < 0.001) and D‐dimer (0.652, 0.600–0.704, *p* < 0.001). USG and D‐dimer levels were further calculated using the optimal cutoff values. The optimal cutoff was 1.019 for USG (+PV = 0.847, −PV = 0.302, +LR = 1.593, −LR = 0.668) and 0.50 for D‐dimer (+PV = 0.911, −PV = 0.300, +LR = 2.958, −LR = 0.673). After propensity score matching, only D‐dimer levels (AUC, 95%CI: 0.703, 0.643–0.764, *p* < 0.001) had an AUC of >0.6. The optimal cutoff for the D‐dimer was 0.50 (+PV = 0.917, −PV = 0.313, +LR = 3.196, −LR = 0.634).

For differentiating CVST from healthy subjects (Table S2), parameters with AUC >0.6 only included fibrinogen (AUC, 95%CI: 0.605, 0.548–0.662, *p* < 0.001). The optimal cut‐off fibrinogen level was 3.445 (+PV = 0.961, −PV = 0.299, +LR = 7.120, −LR = 0.678). After propensity score matching, only fibrinogen (AUC, 95%CI: 0.603, 0.536–0.671, *p* < 0.001) had an AUC >0.6, with an optimal cutoff value of 3.445 (+PV = 0.960, −PV = 0.297, +LR = 7.000, −LR = 0.684).

### 3.5 Association of dehydration‐related indices to CVST severity and prognosis

Spearman's correlation analysis showed that the glucose level had a mild correlation with the baseline NIHSS (*r* = 0.400, *p* < 0.001) and 7‐day NIHSS (*r* = 0.345, *p* < 0.001) scores in patients with CVST. However, other renal function markers were not correlated with baseline NIHSS, baseline mRS, 7‐day NIHSS, or 7‐day mRS scores (all *r* < 0.3; Table 5).

**TABLE 5:** The association of dehydration related indices with the severity and the prognosis of CVST.

Indices	NIHSS at admission	mRS at admission	NIHSS at 7 days	mRS at 7 days
Spearman's rank correlation coefficient, *r*, *p*				
Cr	−0.069, 0.320	−0.162, 0.208	−0.093, 0.208	−0.220, 0.001
Urea	0.057, 0.418	−0.022, 0.750	0.070, 0.313	−0.027, 0.696
USG	0.136, 0.049	0.100, 0.151	0.144, 0.038	0.127, 0.067
Hematocrit, %	−0.183, 0.008	−0.109, 0.118	−0.121, 0.082	−0.114, 0.100
Na^+^	−0.111, 0.109	−0.017, 0.806	−0.046, 0.507	−0.041, 0.560
K^+^	−0.186, 0.007	−0.102, 0.144	−0.154, 0.026	−0.083, 0.234
Glucose	0.400, <0.001	0.282, <0.001	0.345, <0.001	0.162, 0.020
Uric acid	−0.075, 0.279	−0.196, 0.005	−0.086, 0.218	−0.213, 0.002
UA/Cr	0.084, 0.225	0.055, 0.430	0.111, 0.109	0.114, 0.102
D‐dimer	0.294, <0.001	0.201, 0.004	0.228, 0.001	0.058, 0.402
Fibrinogen	0.229, 0.001	0.129, 0.208	0.189, 0.006	0.021, 0.764
Plasma osmolality, mOsm/(kg·H_2_O)	−0.013, 0.875	0.040, 0.566	0.062, 0.374	−0.003, 0.962

Abbreviations: Cr, creatinine; UA, uric acid; USG, Urine specific gravity. * < 0.05, ** < 0.01, *** < 0.001.

## 4 DISCUSSION

This study investigated the association between dehydration and CVST for the first time and found that (1) a high level of USG, especially for USG >1.013, was more common in patients with CVST than in patients with CVST mimics; (2) with CVST phase development, the level of USG, as well as D‐dimer and fibrinogen, gradually decreased close to the levels seen in patients with CVST mimics; and (3) all dehydration‐related markers were underpowered to differentiate CVST from its mimics and healthy populations, and they were not associated with CVST severity and prognosis.

Previously, more attention has been paid to dehydration in patients with AIS.^7^ It is likely that dehydration can cause contraction of the total plasma volume, increase blood viscosity, reduce cardiac output, and retard collateral circulation formation in the brain.^13^, ^14^ Therefore, dehydration is regarded as a risk factor for AIS and is associated with AIS severity and prognosis.^15^ In theory, dehydration promotes thrombus formation not only in the arteries but also in the veins.^5^ Kelly et al. found that dehydration after AIS was strongly and independently correlated to venous thromboembolism.^5^ However, it remains unclear whether dehydration plays an important role in CVST. CVST is caused by a hypercoagulable state, and dehydration can lead to a hypercoagulable state.^5^, ^6^, ^7^ Therefore, we hypothesized that dehydration is correlated with the occurrence, development, and prognosis of CVST.

A total of 645 people were recruited for this study in order to identify a link between dehydration and CVST. In general, USG, UA/Cr, plasma osmolality, and hematocrit levels can reflect dehydration status.^7^ Therefore, we investigated the differences in these indices among the cohorts. We found that the level of USG and the percentage of patients with USG >1.03 were significantly higher in patients with CVST than in those with CVST mimics. Even after propensity score matching, the percentage of patients with USG >1.03 was still higher in the CVST group than that in the CVST mimic group. USG is an essential marker for evaluating dehydration status and an independent risk factor for early neurological deterioration (END) in AIS.^16^, ^17^, ^18^ Lin et al. demonstrated that USG‐based hydration could prevent END in patients with AIS.^18^ Therefore, we consider alleviating dehydration based on USG levels as a novel therapeutic strategy for CVST, which should be verified in the future. Furthermore, the concentration of UA was lower in patients with CVST than in those with CVST mimics and healthy populations. Urea levels reflect renal function and nutritional status. Patients with CVST suffer from high intracranial pressure, which causes vomiting and nausea, leading to malnutrition. This seems to explain why UA levels were lower in patients with CVST. UA/Cr and BUN/Cr are acknowledged indicators for assessing dehydration status.^7^ However, the UA/Cr ratio was significantly higher in patients with CVST mimics than in patients with CVST or the healthy population. This result seemed to contrast with the USG results. However, the actual differences in UA/Cr among the three cohorts were small despite statistical significance, and the differences diminished after propensity score matching. Hence, the UA/Cr ratio could not accurately reflect the dehydration status of the CVST patients. Our results showed that the differences in plasma osmolality and Na^+^ concentration between patients with CVST and those with its mimics were statistically significant, even after propensity score matching. However, the differences were too small to guide a clinical diagnosis. Previous studies have found that plasma osmolality is associated with the occurrence, development, and prognosis of AIS.^8^ However, our results did not show a correlation in patients with CVST. Plasma osmolality reflects dehydration status, but it is also affected by abnormal ions, glucose, and proteins. Moreover, unlike direct detection, the plasma osmolality estimated using the equation used in this study may have influenced the final results. Interestingly, the concentrations of Cr and hematocrit levels and the number of patients with hematocrit levels >45% were higher in the healthy population. Patients with CVST and CVST mimics frequently experience nausea, vomiting, and headache, which lead to loss of appetite and even malnutrition, resulting in lower Cr and hematocrit levels >45%.

We also found that as the stage progressed, dehydration‐related markers in patients with CVST gradually approached the levels in patients with CVST mimics. The thrombus in the CVST in the chronic stage is organized and develops into stenosis.^10^ Although patients with CVSS derived from CVST have headaches and vomiting, the symptoms are generally milder than those in patients with acute and subacute CVST. The results indicated that with the development of CVST, dehydration was relieved, and hypercoagulation was retarded. Based on the characteristics of dehydration in CVST, we attempted to identify the diagnostic power of dehydration‐related markers in differentiating CVST, CVST mimics, and healthy populations. However, except for coagulation factors such as D‐dimer and fibrinogen, dehydration‐related markers cannot be used for the diagnosis of CVST. Therefore, dehydration‐related markers were not linked to the severity and prognosis of CVST. Although dehydration‐related markers such as USG, UA/Cr, and plasma osmolality are characteristic of AIS and are capable of predicting the clinical outcomes of AIS, we did not find any correlation between these markers and CVST.

This observational study has several limitations. First, dehydration‐related indices detected in this study were scarce. Some dehydration‐related indices such as urine osmolality and glomerular filtration rate were not assessed in the enrolled participants. Furthermore, some dehydration‐related markers, including USG, Na^+^, K^+^, D‐dimer, and plasma osmolality were not detected in healthy individuals. As in any retrospective observational study, the lack of assessment is a major limitation, especially in rare diseases such as CVST. Second, despite the large sample size in this study, the results still seem to be in doubt because the elevation of USG reflected dehydration status in patients with CVST, but reduction of plasma osmolality and hematocrit seemed to indicate a nondehydration status in these patients. We considered that the differences in plasma osmolality among the three cohorts were too small to reach significance, and the lower hematocrit in CVST was likely to be attributed to malnutrition, but the hypothesis was not accurate enough to form convincing conclusions. However, owing to inconsistent evidence, we cannot confirm that dehydration was observed in patients with CVST. Future multicenter, well‐designed prospective studies with large sample sizes are needed to further verify these conclusions.

## 5 CONCLUSIONS

High levels of USG, especially USG >1.013, were more common in patients with CVST, which might indicate dehydration in CVST, than in patients with CVST mimics; however, the conclusion was not convincing because other dehydration‐related indices were inconsistent. With the development of the CVST phase, USG levels gradually decreased to levels close to those in patients with CVST mimics. No evidence revealed that dehydration‐related indices could differentiate CVST from its mimics and healthy populations or were associated with CVST severity and prognosis.

## CONFLICT OF INTEREST STATEMENT

JD, DL, XZ, XL, YD, XT, ZL, XY, MZ, and RM report no conflicts of interest.

## Supporting information


Appendix S1.

## Data Availability

The data presented in the study are available in the supplemental material of this article. Further inquiries can be directed to the corresponding authors.
