# Supplementary material for: Dehydration in cerebral venous sinus thrombosis
Source: CNS Neurosci Ther. 2024 May 22;30(5):e14760. doi: 10.1111/cns.14760 (PMC11110100; doi:10.1111/cns.14760)
Supplement: Supplementary file 1 — Appendix S1. [file CNS-30-e14760-s001.doc]

**Supplemental Material**

**Table S1.** ROC analysis for differentiating CVST from CVST mimics using biomarkers

| Indices | Before propensity score matching | | | After propensity score matching | | |
| --- | --- | --- | --- | --- | --- | --- |
|  | AUC | 95%CI | p-value | AUC | 95%CI | p-value |
| Cr | 0.509 | 0.455-0.564 | 0.735 | 0.413 | 0.346-0.479 | 0.012 |
| Urea | 0.381 | 0.329-0.433 | <0.001 | 0.383 | 0.317-0.448 | 0.001 |
| USG | 0.637 | 0.585-0.688 | <0.001 | 0.568 | 0.500-0.635 | 0.050 |
| Haematocrit, % | 0.543 | 0.488-0.598 | 0.121 | 0.437 | 0.369-0.504 | 0.067 |
| Na^+^ | 0.374 | 0.322-0.426 | <0.001 | 0.375 | 0.309-0.440 | <0.001 |
| K^+^ | 0.438 | 0.385-0.492 | 0.025 | 0.433 | 0.366-0.500 | 0.053 |
| Glucose | 0.504 | 0.450-0.559 | 0.874 | 0.563 | 0.495-0.631 | 0.069 |
| Uric acid | 0.476 | 0.422-0.531 | 0.390 | 0.398 | 0.331-0.464 | 0.003 |
| UA/Cr | 0.370 | 0.318-0.423 | <0.001 | 0.442 | 0.374-0.510 | 0.093 |
| D-dimer | 0.652 | 0.600-0.704 | <0.001 | 0.703 | 0.643-0.764 | <0.001 |
| Fibrinogen | 0.533 | 0.477-0.589 | 0.233 | 0.554 | 0.485-0.622 | 0.212 |
| Plasma osmolality, mOsm/(kg·H_2_O) | 0.353 | 0.302-0.404 | <0.001 | 0.371 | 0.306-0.436 | <0.001 |
| USG: Urine specific gravity; Cr: creatinine; UA: uric acid. The involved parameters should have between-group differences with p value<0.01. | | | | | | |

**Table S2.** ROC analysis for differentiating CVST from health subjects using biomarkers

| Indices | Before propensity score matching | | | After propensity score matching | | |
| --- | --- | --- | --- | --- | --- | --- |
|  | AUC | 95%CI | p-value | AUC | 95%CI | p-value |
| Cr | 0.376 | 0.322-0.430 | <0.001 | 0.312 | 0.254-0.370 | <0.001 |
| Urea | 0.361 | 0.307-0.414 | <0.001 | 0.337 | 0.278-0.397 | <0.001 |
| Haematocrit, % | 0.315 | 0.264-0.366 | <0.001 | 0.249 | 0.197-0.302 | <0.001 |
| Glucose | 0.438 | 0.381-0.494 | 0.029 | 0.446 | 0.378-0.513 | 0.088 |
| Uric acid | 0.431 | 0376-0.487 | 0.016 | 0.385 | 0.323-0.446 | <0.001 |
| UA/Cr | 0.454 | 0.398-0.510 | 0.109 | 0.474 | 0.410-0.539 | 0.423 |
| Fibrinogen | 0.605 | 0.548-0.662 | <0.001 | 0.603 | 0.536-0.671 | 0.001 |
| USG: Urine specific gravity; Cr: creatinine; UA: uric acid. The involved parameters should have between-group differences with p value<0.01. | | | | | | |
